# Visualized SERS Imaging of Single Molecule by Ag/Black Phosphorus Nanosheets

**DOI:** 10.1007/s40820-022-00803-x

**Published:** 2022-03-15

**Authors:** Chenglong Lin, Shunshun Liang, Yusi Peng, Li Long, Yanyan Li, Zhengren Huang, Nguyen Viet Long, Xiaoying Luo, Jianjun Liu, Zhiyuan Li, Yong Yang

**Affiliations:** 1 State Key Laboratory of High-Performance Ceramics and Superfine Microstructures, Shanghai Institute of Ceramics, Chinese Academy of Sciences, 1295 Dingxi Road, Shanghai, 200050 People’s Republic of China; 2 Graduate School of the Chinese Academy of Sciences, No. 19(A) Yuquan Road, Beijing, 100049 People’s Republic of China; 3 Center of Materials Science and Optoelectronics Engineering, University of Chinese Academy of Sciences, Beijing, 100049 People’s Republic of China; 4 State Key Laboratory of Oncogenes and Related Genes, Shanghai Cancer Institute, Renji Hospital, Shanghai Jiaotong University School of Medicine, 200032 Shanghai, People’s Republic of China; 5 School of Physics and Optoelectronics, South China University of Technology, Guangzhou, 510641 People’s Republic of China; 6 Department of Electronics and Telecommunications, Saigon University, Hochiminh City, Vietnam

**Keywords:** Black phosphorus, Single molecule, SERS, Exosome, Machine learning

## Abstract

- Ag/BP-NS exhibit remarkable surface-enhanced Raman scattering performance with single-molecule detection ability. This remarkable enhancement can be attributed to the synergistic resonance enhancement of R6G molecular resonance, photo-induced charge transfer resonance and electromagnetic resonance.
- A new polarization-mapping method was proposed, which can quickly screen out single-molecule signals and prove that the obtained spectra are emitted by single molecule.
- The recognition of different tumor exosomes can be realized combining the method of machine learning.

**Abstract:**

Single-molecule detection and imaging are of great value in chemical analysis, biomarker identification and other trace detection fields. However, the localization and visualization of single molecule are still quite a challenge. Here, we report a special-engineered nanostructure of Ag nanoparticles embedded in multi-layer black phosphorus nanosheets (Ag/BP-NS) synthesized by a unique photoreduction method as a surface-enhanced Raman scattering (SERS) sensor. Such a SERS substrate features the lowest detection limit of 10^–20^ mol L^−1^ for R6G, which is due to the three synergistic resonance enhancement of molecular resonance, photo-induced charge transfer resonance and electromagnetic resonance. We propose a polarization-mapping strategy to realize the detection and visualization of single molecule. In addition, combined with machine learning, Ag/BP-NS substrates are capable of recognition of different tumor exosomes, which is meaningful for monitoring and early warning of the cancer. This work provides a reliable strategy for the detection of single molecule and a potential candidate for the practical bio-application of SERS technology. 
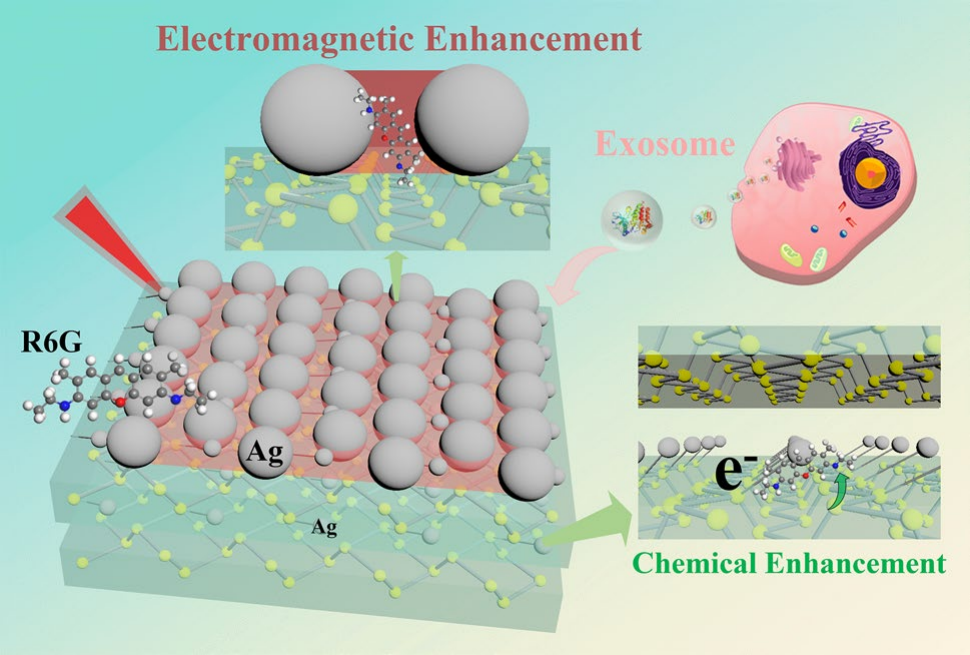

**Supplementary Information:**

The online version contains supplementary material available at 10.1007/s40820-022-00803-x.

## Introduction

Trace detection is of great significance in the fields of environmental science, medical diagnosis, food safety and coronavirus detection [1–5]. With the need of reducing limit of detection (LOD), especially in the detection of biomarkers, the content of target in the clinical sample is usually much low, which may reach to pico-molar, femto-molar or even atto-molar level [6]. For instance, nanovesicles known as exosomes are secreted from a variety of tissues and circulate in biological fluids, which have been implicated in a number of human diseases, including cancer, and are becoming an appreciated fundamental aspect of tumor progression and metastasis [7, 8]. But the tumor exosomes in the blood of patients may be single-vesicle level, which poses a great challenge to the existing detection methods [9]. Surface-enhanced Raman scattering (SERS) is a powerful tool to detect the spectral signals of molecules even at the single-molecular level [10–13]. It is widely used in biochemical analysis, such as pesticide residue analysis, virus detection, tissue tumor recognition, and even bioimaging due to its high sensitivity and molecular specificity [14–18]. However, there are still two obstacles: the fabrication of ultra-sensitive SERS substrates and the detection of single molecule (SM) over the surface of nanostructures, i.e., to achieve a single-molecule SERS-imaging [19–23].

In recent years, two-dimensional (2D) material is a kind of promising SERS substrate due to their high carrier mobility, excellent photoelectricity activity, and large specific surface area [24, 25]. As a new member of the 2D material family, BP nanosheets have good near-infrared absorption and high photothermal conversion efficiency, which has been used for field-effect transistor, high-efficiency photothermal cancer treatment and photoacoustic biological imaging [26–32]. Meanwhile, there are only three Raman fingerprint peaks of black phosphorus (BP) below 500 cm^−1^, far away from the fingerprint area of biological samples (600–1800 cm^−1^), which will not interfere with the signal of biomarkers at all [18]. Therefore, the BP nanosheet is an ideal SERS material. However, the existing research shows that the intrinsic SERS enhancement factor of BP is weak. Kundu et al. formed nano-cavity arrays on BP nanosheets by low-power laser irradiation, and the LOD for R6G was 10 nM [33]. The LOD of BP/Ag and BP/Au/Ag prepared by Li et al. for R6G is 10^–9^ and 10^–12^ M, respectively [34]. Therefore, it is very necessary to develop unique nanostructure boosted by synergistic electromagnetic enhancement (EM) and chemical enhancement (CM) to further improve the SERS sensitivity of BP and its hybrid nanomaterials.

**Scheme 1:**
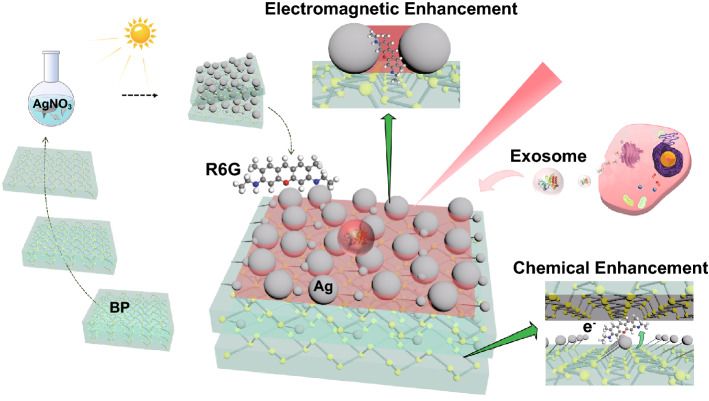
Schematic diagram of the synthesis and application of Ag/BP-NS SERS sensor. The silver nanospheres were introduced in situ on the BP nanosheets by a photoreduction method

Here, we report an ultra-sensitive SERS substrate to realize the visualized detection and imaging of a SM by developing special-engineered nanostructure of Ag nanoparticles embedded in multi-layer BP nanosheets (Scheme 1). One unique “multi-layer nut cake”-liked nanostructure of Ag/BP-NS was designed and fabricated by photo-driven chemical reduction of Ag nanoparticles. Besides the presence of large aggregated Ag nanoparticles (ca. 50 nm) as “hot spots” for the EM, many ultra-small Ag nanoparticles (3–5 nm) were tightly attached on the surface and intercalation of BP, which produced the photo-induced charge transfer (PICT) channel for Ag–P-R6G and resulted for huge CM. The substrate can detect a clear signal of single R6G molecule in 10^–20^ M R6G solution, demonstrating one of the highest sensitivities among those reported SERS substrates. Furthermore, we proposed a polarization-mapping method to demonstrate the visualized SERS detection and Raman imaging of single R6G molecule. By analyzing the intensity changes of R6G at different concentrations and the polarization-mapping spectra of R6G, it was proved that the obtained spectra were emitted by SM, realizing the localization and visualization of SM. As a practical application, single tumor exosome can be recognized using the proposed SERS protocol combining with the method of machine learning. The preparation and research of 2D Ag/BP-NS nanohybrids provided a reliable strategy for the detection and SERS-imaging of SM, which has an excellent biological application prospect.

## Materials and Methods

### Materials

The BP crystals (99.998%) were purchased from XFNANO. Silver nitrate (99.00%), sodium citrate (99.00%), ethanol (99.8%) and rhodamine 6G (99.00%) were purchased from Aladdin. Fetal bovine serum (Foundation FBS, American origin, 900–108) was purchased from Gemini. Penicillin/streptomycin (15,140,122) and RPMI Medium 1640 basic (8,120,141) were purchased from Gibco. McCoy’s 5A Medium (01–075-1A) was purchased from Biological Industries. Total exosome isolation reagent (4,478,359, from cell culture media) was obtained from Thermo Fisher Scientific. And PBS was purchased from BOSTER. All the chemicals were used as received without further purification. Glassware was rinsed with deionized water and ethanol absolute several times prior to experiments.

### Synthesis of Black Phosphorus

The BP nanosheets were prepared with an ultrasonic horn followed by ice bath sonication of the ground powders of bulk BP in Argon. 50 mg bulk BP crystal was dispersed 100 mL deionized water, which has continuously bubbled with Argon for 5 min to eliminate O_2_. After bubbling treatment, the bulk BP was sonicated under the protection of argon for 60 h in an ice bath to get ultra-thin BP nanosheets. The obtained BP nanosheets supernatant was centrifuged at the rotational speed of 5000 r min^−1^, and the supernatant was stored in argon atmosphere at 4 ℃ for further use.

### Synthesis of Black Phosphorus-Ag Nanosheets (Ag/BP-NS)

Silver nanoparticles were prepared by in situ chemical reduction on the surface of BP nanosheets. 10 mL BP nanosheets dispersion synthesized above was dispersed in a mixture containing 40 mL of deionized water and 2 mL AgNO_3_ (0.5 g/100 mL). Subsequently, the solution reacted for 30 min with fluorescent lamps. Here, the light source used a xenon lamp to simulate sunlight. Finally, the solid was collected by centrifuged at 12,000 r min^−1^ for 15 min and washed with deionized water three times to remove the free Ag nanoparticles.

### Cell Culture and Extraction of Exosomes

#### Cell Culture

Fetal bovine serum used in this experiment was previously depleted of exosomes by centrifugation with 120,000 × g, 16 h. All cell lines were purchased from the American Type Culture Collection (ATCC). A549 cell line (Human lung carcinoma) was cultured in RPMI Medium 1640 basic with 5% FBS, 1% penicillin/streptomycin. HCT-116 cell line (Human colorectal carcinoma) was cultured in McCoy’s 5A Medium with 5% FBS, 1% penicillin/streptomycin. All cell lines were cultured in a humidified incubator at 37 ℃ in an atmosphere containing 95% air and 5% CO_2_.

#### Exosome Isolation

The cell culture medium was centrifuged at the rate of 2000 × g for 30 min to remove cells and debris. Then, the exosome separation reagent and the obtained cell-free culture media were mixed in a volume ratio of 1:2. The mixture was incubated overnight at 4 ℃. The incubated samples were centrifuged at 4 ℃ for 1 h at 10,000 × g. Finally, the precipitated exosomes were dispersed in an appropriate volume of 1X PBS. The concentration of exosomes extracted and purified from cell culture medium was 1 × 10^9^ particles mL^−1^, and the solvent was PBS buffer solution.

### Apparatus

The xenon lamp is made by PerfectLight, and working current is 20 A. The SEM images of BP and Ag/BP-NS were obtained by high-resolution field emission scanning electron microscope (Verios G4) at an accelerating voltage of 10 kV. The transmission electron microscopy (TEM), high-resolution TEM (HRTEM), energy-dispersive X-ray spectroscopy (EDS) and selected area electron diffraction (SAED) images were obtained on a JEM-2100F field emission transmission electron microscope at an accelerating voltage of 200 kV. The X-ray diffraction (XRD) measurements were made using a Bruker D8 Discover high-resolution X-ray diffractometer (parameters: Cu *K*_*α*_ radiation,$\lambda = 1.54$, 40 mA and 40 kV). The XPS images were obtained by the Thermo Fisher Scientific (ESCAlab250) X-ray photoelectron spectrometer. The atomic force microscopy (AFM) images were measured by a NT-MDT atomic force microscope (NTEGRA). And the UV–vis images were obtained by a PerkinElmer Ultraviolet visible spectrophotometer (Lambda 950).

### SERS Measurements

The R6G aqueous solution with the different concentrations of 10^–7^-10^–20^ M was used to investigate the SERS performance of BP and Ag/BP-NS. To explore the SERS detection ability of Ag/BP-NS substrates for tumor exosomes (1 × 10^9^–5 × 10^7^ particles mL^−1^), Raman spectra of the A549 and HCT116 exosomes were detected. For each test, the 1.25 mg of synthesized Ag/BP-NS mixed with 50 μ $\mathrm{L}$ (10^–7^–10^–18^ M) R6G aqueous solution or mixed with 100 μ $\mathrm{L}$ exosomes. Subsequently, the mixture was ultrasonicated for 10 min. Finally, the mixture was dripped onto a clean glass slide with a micro-pipettor and dried in an oven at 40 ℃. The liquid diffusion area was about (π × 0.5^2^) cm^2^. For each mapping scanning, the area is a 60 × 60 μm^2^ (100 points) rectangle every time. All the Raman spectra were obtained from Renishaw in Via Reflex laser confocal micro-Raman spectrometer at light wavelength of 532 nm (1.2 mW, 10 s) and 633 nm (0.6 mW, 1 s) for R6G molecules and tumor exosomes, respectively. And the laser beam was focused on a spot about 2 μm in diameter.

## Results and Discussion

### Substrate Characterizations

**Fig. 1:**
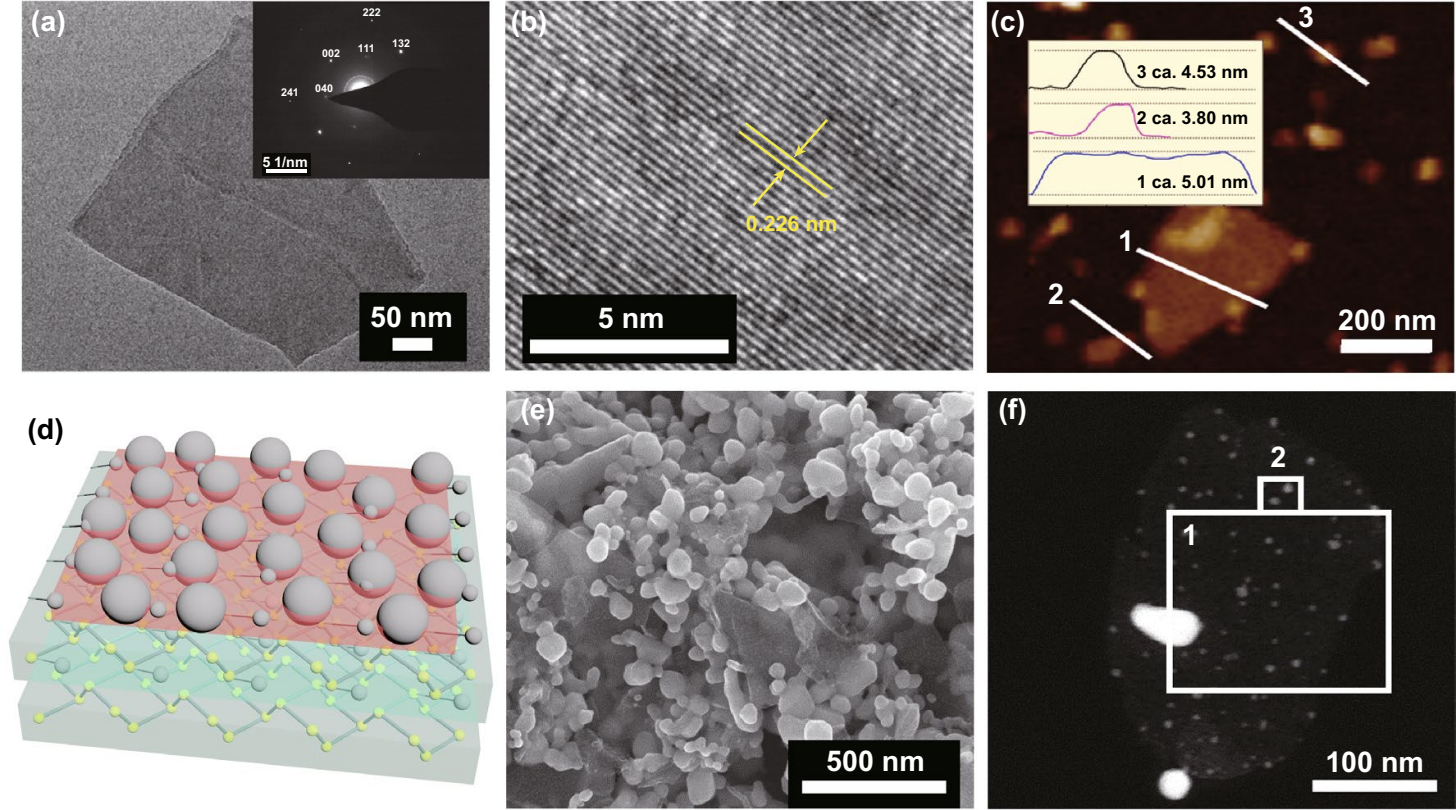
Characterizations of the BP and Ag/BP-NS. **a** TEM bright field image of the BP nanosheets nanostructure. Inset shows the SAED pattern of the BP nanosheets. **b** HRTEM image of single BP nanosheet nanostructure, showing clear lattice fringe with a spacing of 0.226 nm. **c** AFM height image of BP nanosheets with different sizes. Inset shows the AFM height image corresponding to the line. **d** Schematic of the Ag/BP-NS synthesized by photoreduction. **e** SEM and **f** ACTEM high-angle annular dark-field image of the Ag/BP-NS synthesized by photoreduction

The morphology and structure of BP are displayed in Figs. 1 and S1. Under the SEM and TEM, the transverse size of the BP nanosheets is about a few hundred nanometers (Fig. 1a). The inset shows the selective area electron diffraction (SAED) pattern of the BP nanosheets. The BP nanostructure is confirmed to be a single-crystal structure with (111), (002), (132), and (241) diffraction spots of orthorhombic BP (PDF#76–1957). And the high-resolution transmission electron microscope (HRTEM) is shown in Fig. 1b. The HRTEM image displays clear lattice fringes with interplanar spacing of 0.226 nm, which correspond to the (041) crystal plane. In order to characterize the thickness of these nanosheets, AFM images were obtained. As shown in Fig. 1c, the typical thickness of these nanosheets is 3–5 nm about 2–4 layers. The measured thickness of the monolayer is slightly thicker than the theoretical thickness, maybe resulted from the absorption of chemical groups (such as H_2_O or O_2_ molecules) on the oxidized surface of BP nanosheets [35].

Based on the BP nanosheets prepared above as raw materials, we synthesized Ag/Black Phosphorus nanosheets (Ag/BP-NS) under strong illumination. Photoreduction is essential in substrate preparation. As shown in Fig. 1d, it is worth noting that the hybrid nanosheet synthesized in this work is significantly different from the reported Ag/BP-NS composites [34]. Besides large Ag nanoparticles on the surface, there are also ultra-small Ag nanoparticles between the surface and intercalation of BP nanosheets. As shown in Fig. 1e, most of the Ag nanoparticles are well embedded on BP nanosheets. The size of these Ag nanoparticles is about 50–100 nm. Subsequently, we diluted the Ag/BP-NS suspension and peeled off the nanosheets to expose the inner layer. As shown in the high-angle annular dark-field (HAADF) image (Fig. 1f), it is observed that a large number of uniform fine Ag nanoparticles with a diameter of about 3–5 nm are tightly attached on the surface or intercalation of BP nanosheets. However, almost no small silver nanoparticles with the size of 3–5 nm are observed in the Ag/BP-NS samples synthesized without illumination (Fig. S1a, b). It should originate from that the BP nanosheets can generate a large number of photogenerated electrons under the light-driven excitation [36], which makes the nucleation speed of Ag nanoparticles increase rapidly, thereby improving the reduction efficiency of the Ag nanoparticles in the solution [37]. Some nucleated nanoparticles will not be able to grow before the depletion of Ag, so the ultra-small Ag nanoparticles are deposited between the surface and intercalation of the nanosheets in situ. The local elemental mappings of the Ag/BP-NS nanostructure in region 1 and region 2 are shown in Fig. S1c, d. The P, Ag, and O elements are distributed within the entire nanosheet in region 1 of Fig. 1f. Especially the element of Ag in Fig. S1d corresponds to the small bright spots in the HAADF image of region 2 (Fig. 1f). For further analysis of microstructure and the state of the elements in the nanosheets, please refer to Figs. S2 and S3 and the corresponding explanation in S1.

### Single-molecule Detection on Ag/Black Phosphorus Nanosheets

Here, rhodamine 6G (R6G) is used as the probe molecule to study the Raman enhancement properties of Ag/BP-NS. The Raman spectrum of R6G powder is shown in Fig. S4, in which the chemical bond vibrations corresponding to Raman shifts are listed in Table S1. The Raman shifts at 854, 1360, and 1648 cm^−1^ are mainly attributed to the C–H deformation, xanthene ring stretch and in plane C–H bend of xanthene, respectively [38–40]. Subsequently, we systematically studied the SERS properties of these Ag/BP-NS. The results are displayed in Figs. S5 and 2. Both BP nanosheets and Ag/BP-NS perform strong SERS enhancement for probe molecules.

**Fig. 2:**
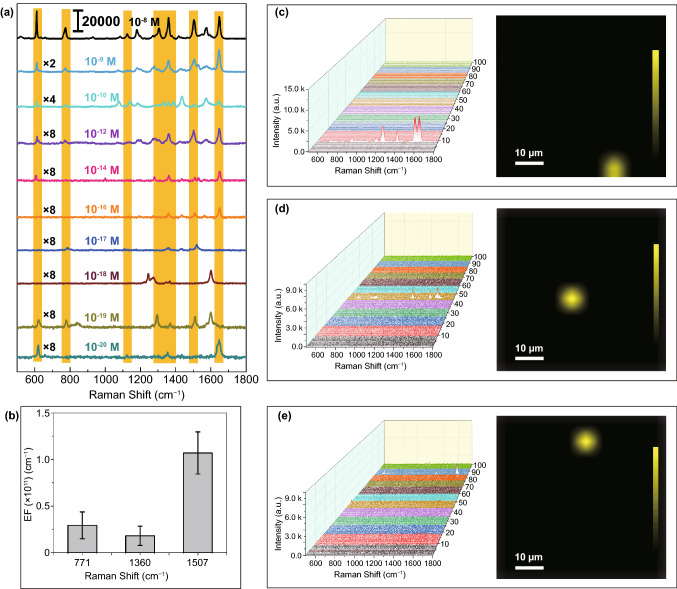
SERS performance and SM imaging characterization of Ag/BP-NS. **a** Raman spectra of R6G with different concentrations of 10^–8^, 10^–9^, 10^–10^, 10^–12^, 10^–14^, 10^–16^, 10^–17^, 10^–18^, 10^–19^, and 10^–20^ M on Ag/BP-NS substrates. The × *n* (*n* = 2, 4, 8) on the left side of the figure is the magnification of the Raman spectra under this scale. **b** The histogram of SERS enhancement factors of single R6G molecule on Ag/BP-NS substrates at 771, 1360, and 1507 cm^−1^. Raman mapping images and SERS-imaging of R6G with different concentrations of **c** 10^–18^ M, **d** 10^–19^ M, and **e** 10^–20^ M on Ag/BP-NS substrates with the area of 60 × 60 μm^2^

The LOD of R6G without Ag modification can reach to 10^–8^ M (Fig. S5a, b) with an enhancement factor (EF) of 6.67 × 10^7^ at 854 cm^−1^ (Fig. S5c), which is comparable to the BP with local hot spots introduced by laser etching or even the BP modified by noble metal [33, 34]. And the LOD for R6G on Ag/BP-NS prepared without illumination is only 10^–9^ M as shown in Fig. S6, which is similar to the results reported in the literature [34]. Most importantly, the Ag/BP-NS fabricated by photoreduction show the extremely high SERS-sensitivity with the LOD of 10^–20^ M as shown in Fig. 2a, demonstrating one of the highest sensitivities among all reported SERS substrates [41–43]. It also indicates that the LOD of Ag/BP-NS synthesized with illumination can reach the level of SM (Please refer to S3 for details). Meanwhile, the maximum EF is 0.101 × 10^12^ at 1507 cm^−1^ at the concentration of 10^–17^ M corresponding to the aromatic C–C stretch (Fig. 2b).

In order to verify the single-molecule detection ability of Ag/BP-NS, the following verification was carried out. In general, the detectable molecule number in micro-Raman measuring area (laser focusing area) can reach the level of SM or several molecules once the concentration of the probe molecules is less than 10^–11^ M (see S4 for details) without the enrichment effect of the probe molecules. As a result, we obtained a series of Raman signals of probe molecules with concentration gradient from 10^–8^ to 10^–20^ M (Fig. 2a). The Raman signal of R6G was obtained from mappings (60 × 60 μm^2^ rectangular region) when the concentration was lower than 10^–13^ M. It should be noted that the fingerprint peaks of R6G will not all appear at low concentrations due to the polarization characteristics of single molecule. And only vibrations with the same polarization direction as the polarization direction will be enhanced. Those Raman mapping images and SERS-imaging of R6G with different concentrations of 10^–16^ ~ 10^–20^ M on Ag/BP-NS substrates are shown in Figs. S7a-d and 2c-e. After several mapping scans of different regions, the highest fingerprint peak in the range of 1500–1700 cm^−1^ was selected for SERS-imaging in the area with signal. As a result, only 1–3 isolated signals can be obtained in each measured area, which are regarded as SM emitting.

**Fig. 3:**
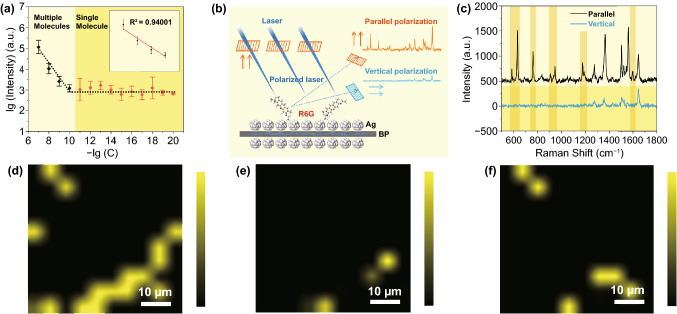
Characterization of single molecule. **a** Raman intensity of R6G at 1360 cm^−1^ as a function of the R6G concentration. Inset shows the enlarged spectrum in the range of 10^–7^ – 10^–10^ M. **b** Schematic of the SM polarized Raman spectra on Ag/BP-NS. **c** Polarized Raman spectra of R6G single molecule. **d** Mapping spectrum of single R6G molecule and polarization-mapping spectra in (**e**) parallel and (**f**) vertical directions

In fact, not every scanning area can find the molecule. For example, the signal collection of R6G solution of 10^–18^ M in a larger region (100 points × 10, 0.77 × 0.48 mm^2^) is shown in Fig. S7e-f, and three single-molecule signals are observed. At such a low concentration, we can rule out the interference of molecular aggregation, which can be considered that we really get the signal of a SM. Subsequently, the intensity of characteristic peak at 1360 cm^−1^ for R6G on Ag/BP-NS substrate was counted. The linear correlation between the data (Y-axis coordinate is the base-10 logarithm of Raman intensity) was calculated in the range of 1.0 × 10^–7^ to 1 × 10^–20^ M (Fig. 3a). The linear relationship is satisfactory in the range of 10^–7^ to 10^–10^ M with the correlation coefficient of 0.94001. However, when the concentration is lower than 10^–11^ M, the probe signal obtained is no longer correlated with the previous data. And the intensity fluctuation between the data changes little, indicating that there are only one or several R6G molecules at the measured point where the signal appears.

Then, we proposed a polarization-mapping method to explore whether the R6G signal obtained from ultra-low concentration samples was emitted by SM. Figure 3b shows a schematic diagram of the polarization strategy. The SM of R6G is asymmetric, and the molecule has polarity. Therefore, its Raman spectrum has polarization characteristics [44]. If the Raman spectrum is not emitted by SM but a large number of molecules are adsorbed on the substrate and randomly distributed. The polarization tensor of the collected molecular signal in each direction is the statistical result with average effect. Therefore, when a polarizer is added to the optical path, there will only be a difference in intensity between the polarization spectra [44, 45]. On the contrary, for the single-molecule polarization spectra with polarizers in different directions in the optical path, their differences will not only display in the intensity, but will also lead to the disappearance and the change of relative intensity of some peaks. Therefore, whether the signal was emitted by SM can be judged by studying the polarized Raman spectra of R6G molecules.

Here, a 60 × 60 μm^2^ rectangular region was scanned in 10^–15^ M R6G samples by mapping and analyzed the clear signal in it. We placed a polarizer behind the laser and in front of signal collector, respectively. Then, we turned the polarizer in front of the signal collector to make it parallel or vertical with the polarizer behind the laser. The parallel and vertical polarization spectra of R6G were collected at the same measured point. As mentioned in Fig. S2b, BP has three intrinsic Raman peaks at 364, 440, and 468 cm^−1^, which can be used as an ideal control group. As shown in Fig. S8a, we randomly selected the fingerprint peaks of BP at some points in the mapping results as a comparison. For the Ag/BP-NS substrate, there was only the difference of peak intensity between vertical polarization spectrum and parallel polarization spectrum, and there was no peak disappearance or the change of relative intensity between each peak. As shown in Figs. 3c and S8b, we also performed some analysis for the R6G molecule. However, after rotating the polarizer in front of the signal collector by 90°, there was an obvious disappearance of some fingerprint peaks and the change of relative intensity between the parallel polarization spectrum and the vertical polarization spectrum. It was due to the polarizer filtering out the scattered photons of SM in other directions, which is the important evidence of the existence of SM [44, 46]. Based on the above principles and experimental results, we proposed a method combining mapping and polarization spectra to characterize SM. As shown in Fig. 3d-f, here, we taken the Raman intensity at 1648 cm^−1^ for mapping imaging. There were 16 clear molecular signals obtained when no polarizer was added in the optical path (Fig. 3d). After adding polarizers to the optical path, the change of the fingerprint peak was reflected in the polarization-mapping spectra. As shown in Fig. 3e, f, some of the clear signals that originally appeared in normal mapping disappeared, and the position of signal cancelation would change after rotating the polarizer. But no matter the parallel polarization spectrum or the vertical polarization spectrum, the position of the signal can be one-to-one corresponding to the spectrum without polarizer. The above phenomena demonstrate that we have realized the visualized detection and characterization of SM based on Ag/BP-NS.

### SERS Enhancement Mechanism

**Fig. 4:**
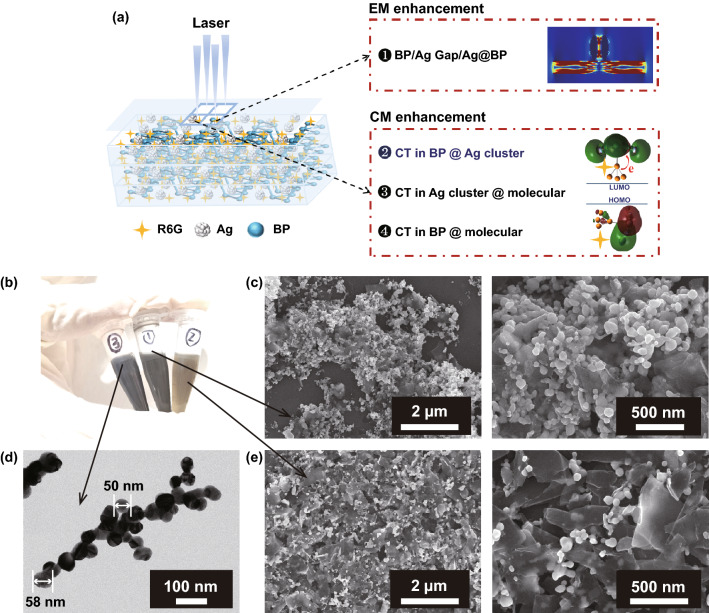
Schematic of synergistic resonance enhancement and the morphology of SERS substrate prepared by different methods. **a** Schematic of the multiple SERS enhancement effects of Ag/BP-NS. **b** The suspension of Ag/NS prepared by (1) photoreduction method and (2) ordinary method and (3) Ag colloids prepared by chemical reduction. **c** The surface morphology of Ag/BP-NS prepared by photoreduction method at different magnifications. **d** TEM bright field image of Ag colloids prepared by chemical reduction. **e** The surface morphology of Ag/BP-NS prepared without illumination at different magnifications

According to the “Unified View” presented by Lombardi et al. [47–49], the high SERS sensitivity of Ag/BP-NS can be attributed to the synergistic resonance enhancement of electromagnetic resonance around the “hot spots” of Ag nanoparticles, photo-induced charge transfer resonance (band gap resonance) and R6G molecular resonance under the 532 nm laser excitation (Fig. 4a). As shown in Fig. 4b, we synthesized the suspension of Ag/BP-NS by (1) photoreduction method and (2) ordinary method and (3) Ag colloids by chemical reduction. The size of Ag nanoparticles synthesized by different methods is similar (Fig. 4b–e). However, the appearance of the hybrid nanosheet synthesized by photoreduction is more similar to that of Ag colloids (more Ag nanoparticles), which is also confirmed by SEM images (Fig. 4c, d). As we mentioned above, the BP nanosheets can produce a large number of photogenerated electrons under the excitation light to improve the reduction efficiency of the Ag nanoparticles. Therefore, the photoreduction method brings higher reduction efficiency like chemical reduction, which will inevitably bring more “hot spots.”

**Fig. 5:**
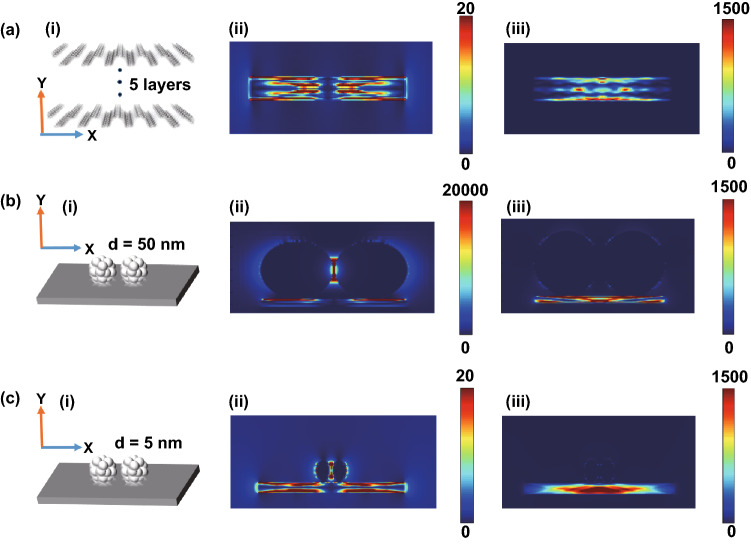
The electromagnetic field simulation results of Ag/BP-NS. **a** (i) Schematic of BP layers with the simulated x–y plane electric field intensity (|E|^2^) along (ii) X polarization and (iii) Y polarization. **b** (i) Schematic of BP layers combined with silver nanoparticles (50 nm) and the simulated x–y plane electric field intensity (|E|^2^) of Ag nanoparticles along (ii) X polarization and (iii) Y polarization, respectively. **c** (i) Schematic of BP layers combined with silver nanoparticles (5 nm) and the simulated x–y plane electric field intensity (|E|^2^) of Ag nanoparticles along (ii) X polarization and (iii) Y polarization

**Fig. 6:**
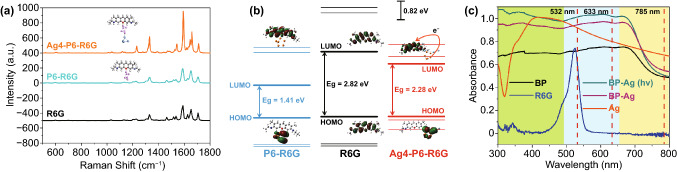
Gaussian simulations of the chemical enhancement and band gap change of BP and Ag/BP-NS substrates. **a** Calculated Raman spectral of R6G molecule, P6-R6G and Ag4-P6-R6G clusters, respectively. **b** Calculated band gap of R6G molecule, P6-R6G, and Ag4-P6-R6G clusters. **c** UV–vis absorption spectra of R6G, Ag colloids, BP, Ag/BP-NS (without illumination), and Ag/BP-NS (with illumination)

As shown in Fig. 5a–c, the electromagnetic field distribution of BP nanosheets and hybrid systems was simulated by finite-difference time-domain (FDTD, details for S5). Localized plasmon resonances of BP can be excited with an incident light (Fig. 4a). Subsequently, we calculated the dimer (Ag nanoparticles) adsorbed on the 5 layers of BP. As shown in Fig. 5b, the Ag particles with the diameter of 50 nm provide obvious electromagnetic enhancement. The strong coupling resonance was mainly distributed in the “gap” of the dimer (Ag nanoparticles) and the surface of BP. The simulated x–y plane electric field intensity (|E|^2^) along **x** polarization (Fig. 5b(ii)) had a maximum intensity (|E|^2^ = 20,000). According to the corresponding relationship between SERS enhancement factor and local electric field enhancement factor, SERS enhancement factor is approximately equal to the fourth power of local electric field enhancement factor [50]. Therefore, in Ag/BP-NS, electromagnetic can provide about 10^8^ enhancement. Although the Ag nanoparticles with the diameter of 5 nm did not provide obvious electromagnetic enhancement (Fig. 5c), the highly efficient carrier mobility in BP/Ag heterostructure can effectively promote the chemical enhancement process and photoactivity [27, 51]. As shown in Fig. 6a, the R6G spectra calculated by Gaussian show that there is an obvious chemical enhancement in Ag4-P6 clusters (S2 for details). More importantly, the calculated band gap of Ag4-P6-R6G clusters is close to the “band gap resonance” (2.33 eV) under 532 nm laser (Fig. 6b).

Besides the calculated results of EM and CM, we have further proved that there was a significant charge transfer resonance in the substrate by experiment. As shown in Figs. S9a and 6c, Ag nanoparticles (ca. 50 nm) strongly enhanced the Raman signal of molecular due to the coupling between the plasmon resonance and the light absorption of R6G at 532 nm. The light absorption of the Ag nanoparticles gradually decreased after 532 nm (Fig. 6c). It was speculated that the enhancement effect will gradually decrease at 633 and 785 nm. As shown in Fig. S9b, the experimental results were indeed consistent with the predicted results. Although the light absorption of Ag/BP-NS decreased rapidly at 785 nm, it did not decrease at 633 nm. However, after changing the excitation wavelength, the enhancement of the substrate disappeared rapidly (Fig. S9c, d), which did not conform to the law of EM. Hence, there must be a CM by “band gap resonance” in the Ag/BP-NS.

Compared with the nanosheets prepared without illumination, the Ag/BP-NS prepared by photoreduction still retain a certain enhancement under the excitation light of 633 nm, which also confirms that photoreduction can bring more “hot spots” to the nanosheets. Moreover, the light absorption intensity of the photoreduction substrate is also significantly higher than that of others. In summary, the Ag/BP-NS prepared by photoreduction possess the synergistic resonance of three components including electromagnetic resonance around Ag nanoparticles (ca. 50 nm), photo-induced charge transfer resonance (Ag–P-R6G cluster) and molecular resonance coupling resonance under the excitation light of 532 nm.

### Tumor Exosomes Detection based on Ag/Black Phosphorus Nanosheets

Exosomes are derived from micro-vesicles formed by the invagination of lysosomal particles in cells [52–54]. They carry the chemical information of nucleic acids, proteins, lipids and metabolites that reflects the characteristics of cells [9, 55]. Therefore, many studies use the exosomes as biomarkers for tumor diagnosis and prognosis. However, traditional detection methods including western blot or enzyme-linked immunosorbent assay (ELISA) require complicated procedures or a large number of samples for detection [56]. In view of these cumbersome operations or limited accuracy limitations, traditional exosomes analysis methods have many obstacles in clinical diagnosis. But the SERS may be an ideal detection method because of its fast and sensitive detection characteristics. Therefore, we tested the application value of prepared Ag/BP-NS in tumor detection due to its high SERS-sensitivity, large specific surface area and excellent biocompatibility. Here, we selected two tumor exosomes from A549 lung cancer cell and HCT116 colon cancer cell for analysis, which are two common human tumor cells [57, 58].

**Fig. 7:**
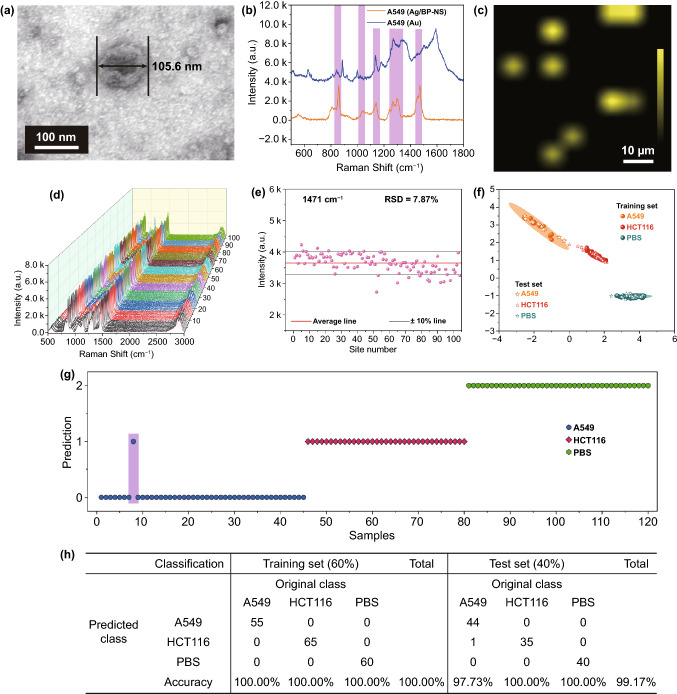
Exosome recognition based on Ag/BP-NS and automatic identification of exosomes by machine learning. **a** TEM image of A549 exosomes. **b** Raman spectra of A549 exosomes with concentration 1 × 10^9^ particles mL^−1^ on Ag/BP-NS and commercial gold substrate. **c** Single A549 exosome detection by SERS-imaging with the concentration of 5 × 10^7^ particles mL^−1^. **d** Raman mapping image of A549 exosomes with concentration 1 × 10^9^ particles mL^−1^. **e** The Raman signal intensities at 1471 cm^−1^ of A549 exosomes in the area shown in Fig. 7d. **f** The training set and test set with 95% confidence ellipses to distinguish A549, HC116, and PBS in two-dimension space by SVM. **g** Predicted labels of test set for A549, HCT116 exosomes and PBS. Value 0, 1, 2 is the prediction as A549 exosomes, HCT116 exosomes and PBS sample, respectively. **h** Confusion matrix of A549 exosomes, HCT116 exosomes and PBS

As shown in Fig. 7a, the size of exosomes is suitable for attaching to Ag/BP-NS. Compared with commercial gold SERS substrate, Ag/BP-NS substrates can well reproduce the fingerprint peaks of A549 exosomes (refer to Table S2 for peak assignment) and show better peaks resolution, indicating the accuracy of identification for exosomes (Fig. 7b). Subsequently, we reduced the concentration of A549 exosomes to 5 × 10^7^ particles mL^−1^. Theoretically, there are only 0.05 exosomes in each Raman detection window (S4 for details). Therefore, there can be only one exosome or no exosomes under each window. As shown in Figs. S10a and 7c, only 9 clear exosome signals were found in the 100 measurement points (60 × 60 μm^2^) mapping). Therefore, we can accurately locate the position of exosomes by SERS-imaging and achieve single-exosome detection.

As we mentioned above, the prepared Ag/BP-NS have uniform SERS enhancement capabilities. The 1 × 10^9^ particles mL^−1^ A549 exosomes adsorbed on Ag/BP-NS substrate were scanned (Fig. 7d). The signal of exosomes was uniform without obvious fluctuation. And the relative standard deviation (RSD) of peak intensity was only 7.87% (Fig. 7e). Thus, the high uniformity of SERS enhancement of exosomes on the substrate indicates that Ag/BP-NS substrate is a potential candidate for the practical application of biological detection. In order to better satisfy the detection requires in the practical application, we also verified the ability of substrate to distinguish different biomarkers. As shown in Fig. S10b, Raman spectra of HCT116 (1 × 10^9^ particles mL^−1^) exosomes and PBS were obtained on Ag/BP-NS substrates. It can be found that PBS does not interfere with the Raman spectra of exosomes at all. However, the Raman spectra of HCT116 and A549 exosomes cannot be easily distinguished only by human eyes. Because the surface composition of the two exosomes is similar, and the difference only lies in the different expressions of certain proteins on the surface [55, 59]. The Raman spectra only show the difference between individual peak (e.g., 1267.4 cm^−1^) and relative intensity of fingerprint peak. Therefore, we used machine learning method to automatically determine and identify different Raman spectra (details for S6) for more intuitively distinguishing different exosomes.

A549, HCT116 two exosomes and PBS interference select 100 spectra for training and testing. Then randomly select 60% of the 300 spectra as the training set, and the remaining spectra as the test set. As shown in Fig. 7f, we reduced the dimension of Raman spectrum to a two-dimensional (2D) space by support vector machine (SVM). It can be found that the signals of A549 and HCT116 exosomes can be clearly distinguished in 2D plane, and their 95% confidence ellipses did not overlap at all. The interference of PBS can be completely eliminated. As shown in Figs. 7g and 7h, the trained model achieved values of sensitivity in the prediction of training and test set equal to 100 and 99.17%, respectively. In fact, only one case of misjudgment appeared in the test set, which shows that Ag/BP-NS have the ability to distinguish tumor exosomes at the single-vesicle level through the machine learning method.

## Conclusion

In summary, we have synthesized a unique “multi-layer nut cake”-liked Ag/BP-NS through a photoreduction method. The Ag/BP-NS show amazing SERS sensitivity with the EF of 0.101 × 10^12^, and the LOD of R6G can reach the single-molecule level. Without any physical enrichment, the clear SM signal can be obtained in 10^–20^ M R6G solution. The excellent SERS enhancement ability of Ag/BP-NS substrate comes from the synergistic resonance enhancement of electromagnetic resonance, photo-induced charge transfer resonance and R6G molecular resonance. Moreover, we realize the precise localization and SERS-imaging of single R6G molecule on Ag/BP-NS by the proposed polarization-mapping spectra and the variation trend of spectral intensity with concentration. The substrate also has excellent performance in practical applications with good biocompatibility and uniformity. It can be used for the detection of single tumor exosome through SERS-imaging, and the tumor exosomes in different cell lines can be distinguished and identified by support vector machine based on Ag/BP-NS substrates. The prepared 2D Ag/BP-NS with single-molecule detection ability, combined with its excellent performance in the field of tumor therapy, this material is expected to establish a unique tumor detection and treatment system.

## Supplementary Information

Below is the link to the electronic supplementary material.

Supplementary file1 (PDF 2108 kb)
