# Supplementary material for: Visualized SERS Imaging of Single Molecule by Ag/Black Phosphorus Nanosheets
Source: Nanomicro Lett. 2022 Mar 15;14:75. doi: 10.1007/s40820-022-00803-x (PMC8922987; doi:10.1007/s40820-022-00803-x)
Supplement: Supplementary file 1 — Supplementary file1 (PDF 2108 kb) [file 40820_2022_803_MOESM1_ESM.pdf]

Supporting Information for

Visualized SERS Imaging of Single Molecule by Ag/Black Phosphorus Nanosheets

Chenglong Lin[1, 2, 3], Shunshun Liang[4], Yusi Peng[1, 2, 3], Li Long[5], Yanyan Li[1, 2, 3] Zhengren Huang[1, *], Nguyen Viet Long[6], Xiaoying Luo[4], Jianjun Liu[1], Zhiyuan Li[5], Yong Yang[1, 3, *]

[1]State Key Laboratory of High-Performance Ceramics and Superfine Microstructures, Shanghai Institute of Ceramics, Chinese Academy of Sciences, 1295 Dingxi Road, Shanghai 200050, People's Republic of China

[2]Graduate School of the Chinese Academy of Sciences, No.19(A) Yuquan Road, Beijing 100049, People's Republic of China

[3]Center of Materials Science and Optoelectronics Engineering, University of Chinese Academy of Sciences, Beijing 100049, People's Republic of China

[4]State Key Laboratory of Oncogenes and Related Genes, Shanghai Cancer Institute, Renji Hospital, Shanghai Jiaotong University School of Medicine, 200032, Shanghai, People's Republic of China

[5]School of Physics and Optoelectronics, South China University of Technology, Guangzhou 510641, People's Republic of China

[6]Department of electronics and telecommunications, Saigon University, Hochiminh City, Vietnam

*Corresponding author. E-mail: zhrhuang@mail.sic.ac.cn (zhengren Huang); yangyong@mail.sic.ac.cn (Yong Yang)

Supplementary Figures

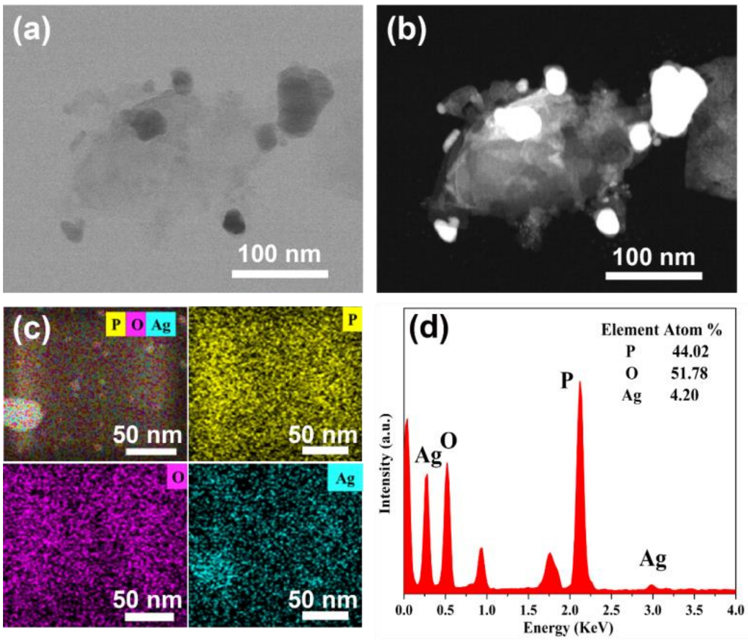

Fig. S1 The morphology of Ag/BP-NS prepared without illumination. (**a**) TEM bright field image and (**b**) TEM high angle annular dark field image of Ag/BP-NS prepared without illumination. (**c**) The local element mappings of the overlay distribution of elements and P, O, Ag in the region 1 of Fig. 1f. (**d**) EDS spectrum of Ag/BP-NS corresponding to the region 2 in Fig. 1f

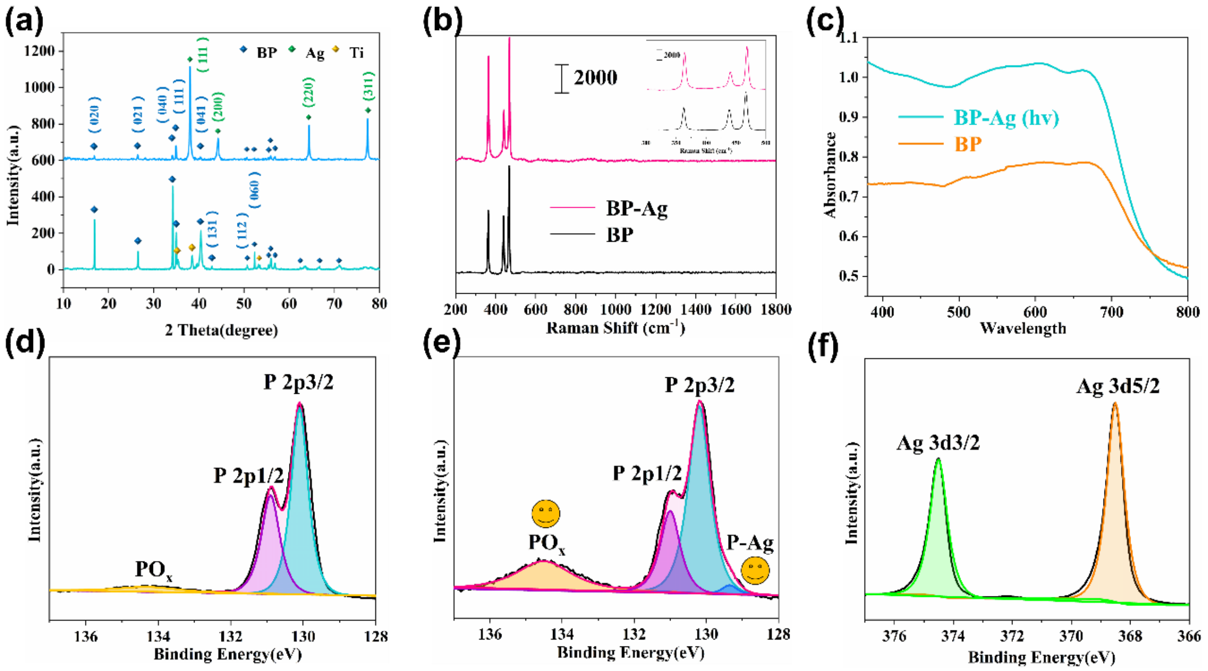

Fig. S2 Characterizations of the BP and Ag/BP-NS. (**a**) XRD patterns of BP nanosheets and Ag/BP-NS. (**b**) Raman spectra of BP nanosheets and Ag/BP-NS, which indicate negligible background signals. Inset shows the enlarged spectra of BP and Ag/BP-NS. (**c**) UV-vis spectra for BP nanosheets compared with Ag/BP-NS. P 2p XPS spectra of the (**d**) BP nanosheets and (**e**) Ag/BP-NS. **f** Ag 3d XPS spectra of the Ag/BP-NS

S1 Characterizations of the BP and Ag/Black Phosphorus Nanosheets

In order to further shed light on the structure of Ag/BP-NS and the state of Ag in the nanosheets, the microstructure and properties of the samples were further analyzed. The X-ray diffraction (XRD), Raman spectra, UV-Vis absorption spectrum and X-ray photoelectron spectroscopy (XPS) are shown in Fig. S2. The XRD results of Ag/BP-NS show that the sample has two sets of lattices, which belong to BP (PDF#76-1957) and Ag (PDF#87-0597) nanostructures (Fig. S2a). It should be explained that a new set of Ti lattice (PDF#89-5009) appeared in BP samples. This is due to the impurity precipitation introduced by the ultrasonic horn of titanium alloy in the sample preparation process, which will not affect the BP nanosheets in the upper layer of the suspension. Figure S2b displays the Raman spectra of the BP nanostructures, in which three distinct fingerprint peaks at about 364, 440, and 468 cm^{-1} can be observed, belonging to one out-of-plane phonon mode ($A^1{}_g$) and two in-plane phonon modes (B_{2g} and $A^2{}_g$), respectively [S1]. We can also notice that there is almost no Raman background in the wavenumber range above 500 cm^{-1} for both BP and Ag/BP-NS, which indicates that Ag/BP-NS is a reasonable choice for SERS substrate for Raman biological fingerprint analysis. The UV-visible absorption spectra of BP and Ag/BP-NS are shown in Fig. S2c. As mentioned above, BP shows abroad light absorption from the entire visible light regions to near-infrared regions. After silver nanoparticles were fabricated on BP nanosheets, the light absorption is obviously enhanced, and the absorption edge is red-shifted. This is due to the local surface plasmon resonance absorption of the surface deposited metal Ag, which results in a significant red shift and broadening of the absorption band of the sample. The extensive light absorption properties of Ag/BP-NS in visible light and near infrared region also show its potential as a material for photothermal therapy of tumors in medicine.

The surface composition and valence of elements in Ag/BP-NS were analyzed by X-ray photoelectron spectroscopy (XPS). The samples were etched by Ar sputtering for 10 seconds to remove the chemical groups adsorbed on the surface. The P, Ag, and O elements in Ag/BP-NS can be observed from XPS spectra. Fig. S2d displays three bands at 130.1, 130.9, and 134.5 eV, which belong to P $2p_{3/2}$, P $2p_{1/2}$ and slightly oxidized phosphorus (P_xO_y) arising from sample processing [S2], respectively. It is worth noting that compared with the black phosphorus without silver nanoparticles, the proportion of the peak of oxidized phosphorus in the BP nanosheets loaded with silver nanoparticles is significantly increased (Fig. S2e), which indicates that the black phosphorus acts as a reducing agent in the in-situ reduction of silver nanoparticles. However, compared with the XPS results of BP and noble metal composites reported in other literatures [S3], the P 2p peak of the composites as-prepared in this study is clear and sharp. At the same time, the peak ratio of oxidized phosphorus peak is obviously lower than that of P 2p, indicating that our hybrid materials are well protected in the process of synthesis and storage, retain their unique two-dimensional structure, and avoid excessive oxidation. In addition, the XPS spectrum of Ag/BP-NS show a sub-band at 129.4 eV, which is attributed to Ag-bonded P [S4]. In Fig. S2f, the bands of 368.5 and 374.5 eV belong to Ag $3d_{5/2}$ and Ag $3d_{3/2}$, respectively. As compared with pure BP and pure Ag, in the case of Ag/BP-NS, the binding energy of P $2p_{3/2}$(130.2 eV), P $2p_{1/2}$(131.0 eV) causes a positive shift of about 0.1 eV, and a positive shift of 0.3 eV for Ag 3d, might result from an intra-atomic CT between the Ag and P atoms. [S5] Our Gaussian calculation results (Fig. S3) also support the charge transfer between silver and phosphorus atoms on the Ag/BP-NS substrate.

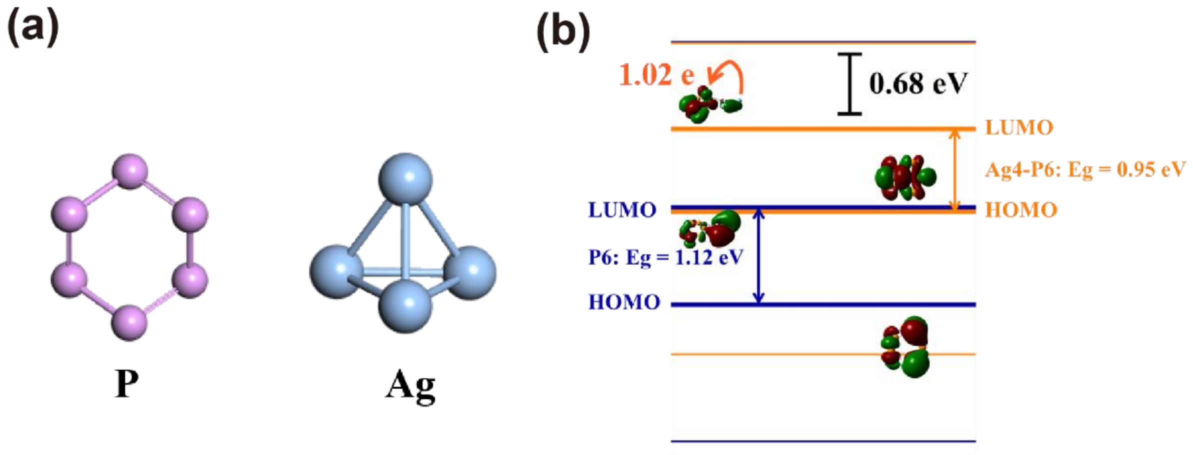

Fig. S3 Charge distribution and surface electrostatic potential distribution of P-Ag cluster. (**a**) The cluster of P and Ag. (**b**) The calculated charge distribution of Ag-P cluster

S2 Calculation Details of Gaussian

The calculated model includes Ag and P atoms. According to the crystal structure of Ag and P, the Ag4-P6 cluster model was constructed to calculate the charge distribution and surface electrostatic potential through Gauss09 program. The 6-311+G (d, p) group including a polarization function and a diffusion function was selected for the P atom in the probe molecules system. The transition metal Ag atom was described by the Lanl2dz basic group.

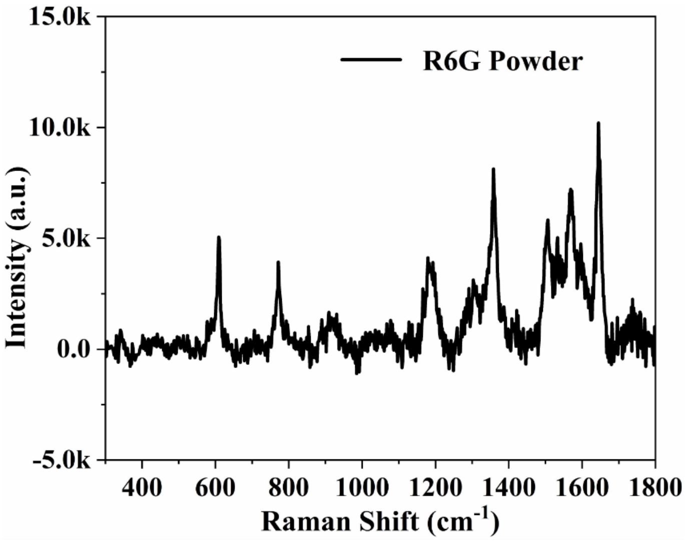

Fig. S4 Raman spectrum of R6G powder

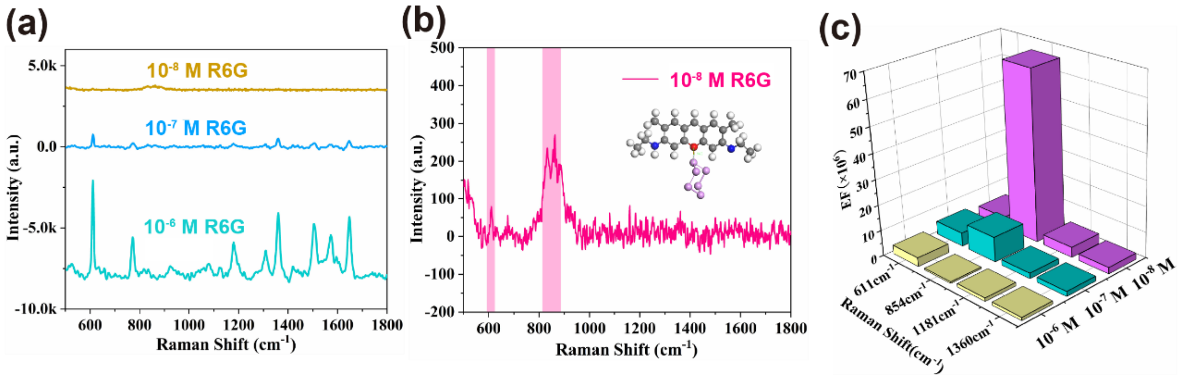

Fig. S5 SERS performance of BP nanosheets. (**a**) Raman spectra of R6G with three different concentrations of 10^{-6}, 10^{-7}, and 10^{-8} M on BP nanosheets substrates. (**b**) The enlarged Raman spectra of 10^{-8} M R6G on BP nanosheets substrates. (**c**) The calculated Raman enhanced factors of R6G with different concentration (10^{-6}, 10^{-7}, and 10^{-8} M) on BP nanosheets at 611, 854, 1181, and 1360 cm^{-1}

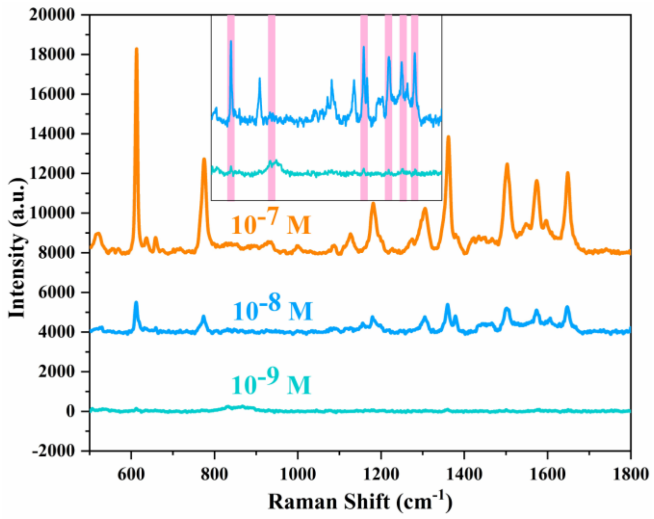

Fig. S6 SERS performance of Ag/BP-NS prepared without illumination. Raman spectra of R6G with different concentrations of 10^{-7}, 10^{-8}, and 10^{-9} M on the Ag/BP-NS substrates prepared without illumination. Inset shows the enlarged Raman spectra of 10^{-8} and 10^{-9} M R6G

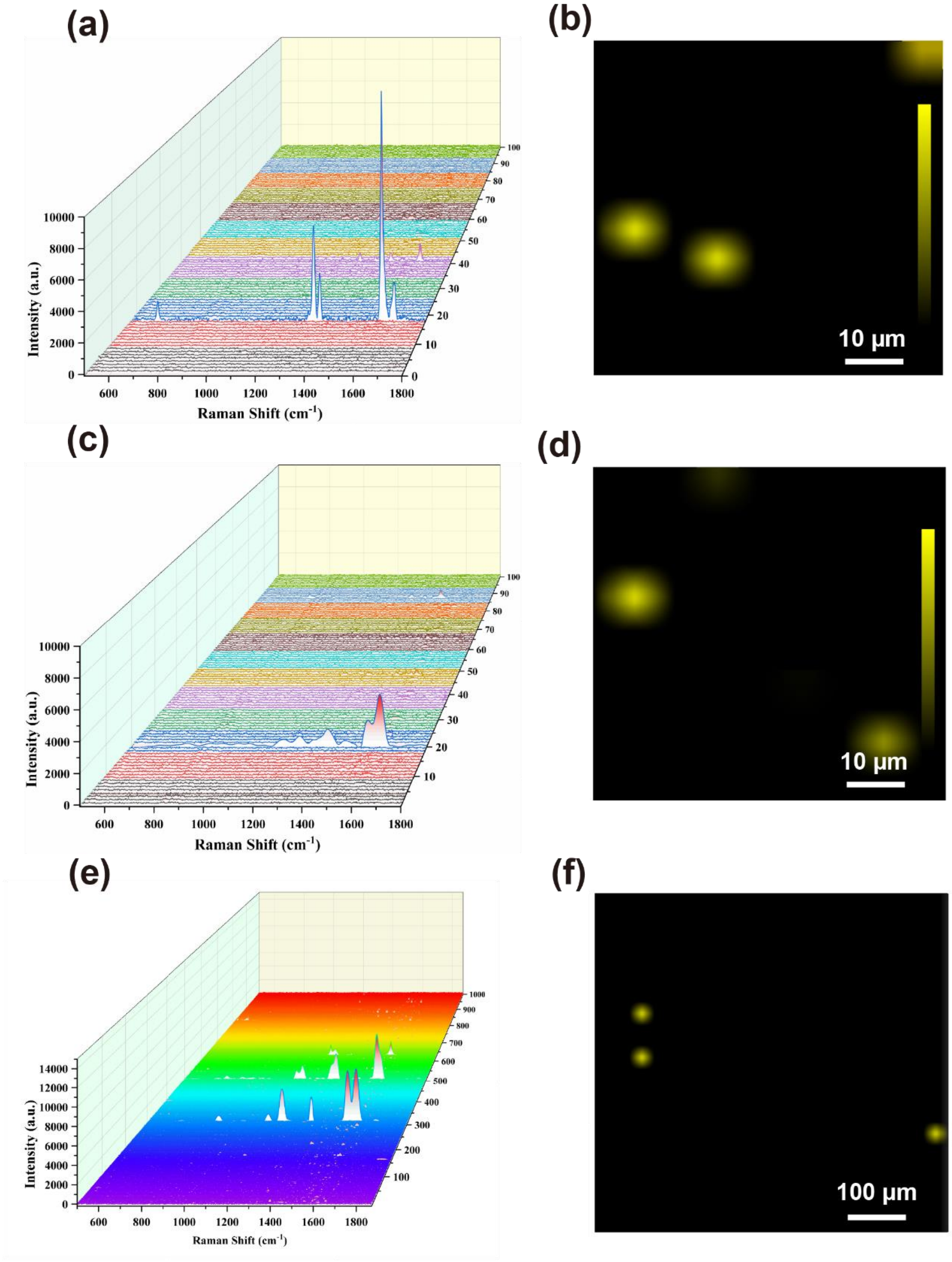

Fig. S7 Raman mapping and SERS-imaging of SM on Ag/BP-NS. (**a**) Raman mapping image and (**b**) SERS-imaging of R6G with the concentrations of 10^{-16} M on Ag/BP-NS substrate with the area of 60 × 60 μm^2. (**c**) Raman mapping image and (**d**) SERS-imaging of R6G with the concentrations of 10^{-17} M on Ag/BP-NS substrate with the area of 60 × 60 μm^2. (**e**) Raman mapping image and (**f**) SERS-imaging of R6G with the concentrations of 10^{-18} M on Ag/BP-NS substrate with the area of 0.60 × 0.60 mm^2

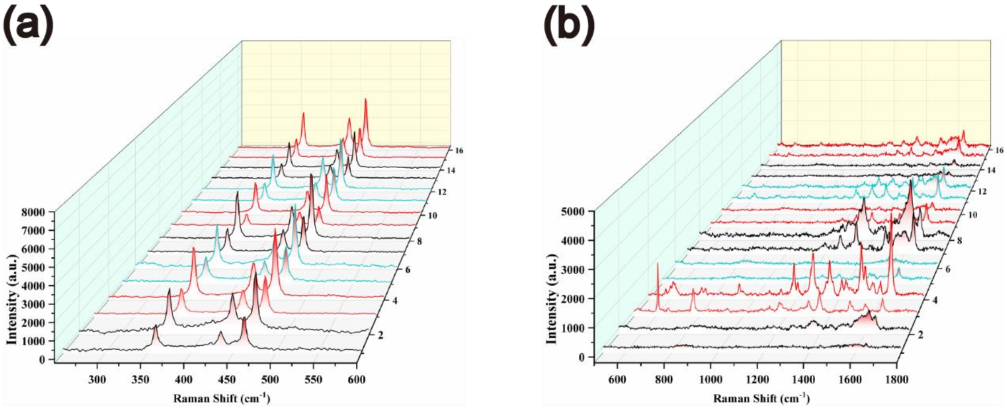

Fig. S8 Polarized-mapping Raman spectra of BP and R6G single molecule. Polarized-mapping Raman spectra of (**a**) BP and (**b**) R6G single molecule. Every two spectral lines with the same color are vertical polarization spectrum and parallel polarization spectrum at the same point, respectively

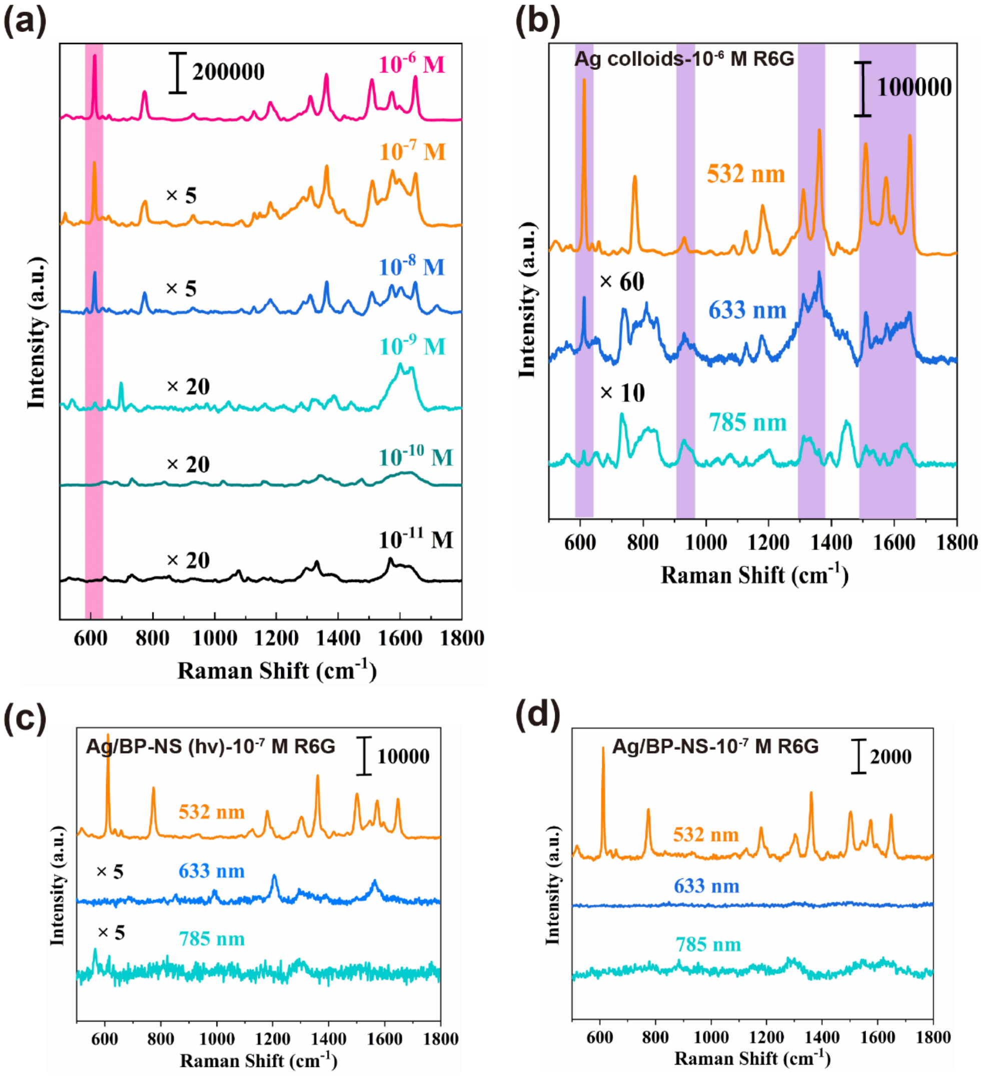

Fig. S9 The enhancement effect of the substrate under different wavelengths of excitation light. (**a**) The enhancement of Ag colloids to different concentrations of R6G. (**b**) The enhancement of Ag colloids to R6G (10^{-6} M) under different excitation wavelengths. (**c**) The enhancement of Ag/BP-NS (with illumination) to R6G (10^{-7} M) under different excitation wavelengths. (**d**) The enhancement of Ag/BP-NS (without illumination) to R6G (10^{-7} M) under different excitation wavelengths

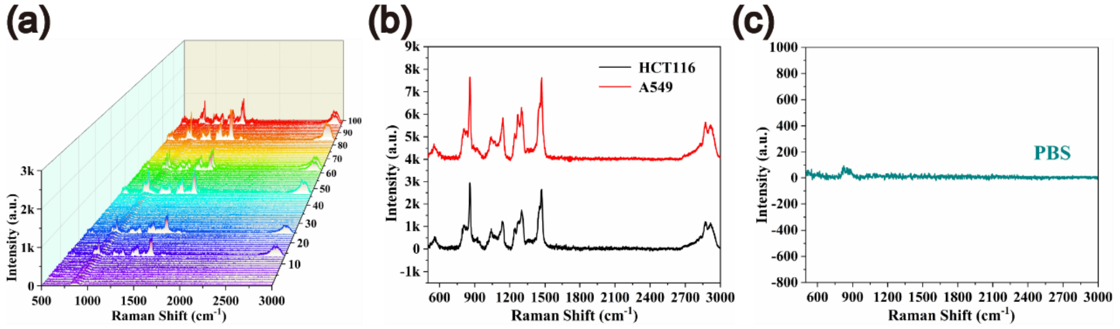

Fig. S10 The Raman spectra of different exosomes and PBS on Ag/BP-NS. (**a**) The Raman spectra of A549 exosomes with concentration 1×10^9 particles/mL on Ag/BP-NS and commercial gold substrate. (**b**) The Raman spectra of A549 and HCT166 exosomes with concentration 1×10^9 particles/ml of on Ag/BP-NS. (**c**) The Raman spectra of PBS on Ag/BP-NS

Table S1 Calculated Raman enhanced factors of R6G with different concentration (10^{-6}, 10^{-7}, and 10^{-8} M) on BP nanosheets at 611, 711, 854, 1181,1360, 1507, 1572, 1600, and 1648 cm^{-1}

Raman Shift (cm^{-1})	Peak Assignment	Enhancement Factor		
		10^{-6} M	10^{-7} M	10^{-8} M
611 cm^{-1}	ρ_{ipb} & ρ_{oopb} of C atoms on Xanthene	3.42×10^6	5.29×10^6	3.99×10^6
771 cm^{-1}	ρ_{ipb} & ρ_{oopb} of C atom on Xanthene	1.61×10^6	3.21×10^6	………
854 cm^{-1}	δ (C-H)	5.96×10^5	9.73×10^6	6.67×10^7
1181 cm^{-1}	δ (C-H)	1.29×10^6	2.09×10^6	3.95×10^6
1360 cm^{-1}	ϕ (C-C)	1.18×10^6	1.86×10^6	2.46×10^6
1507 cm^{-1}	ϕ (C-C)	1.40×10^6	1.98×10^6	………
1572 cm^{-1}	ϕ (C-C)	9.34×10^5	1.27×10^6	………
1600 cm^{-1}	ϕ (C-C)	8.02×10^5	1.75×10^6	………
1648 cm^{-1}	ϕ (C-C)	9.00×10^5	1.30×10^6	………

Abbreviation: δ, deformation; ϕ, aromatic ring; ρ_{ipb}, *in-plane deformation*; ρ_{oopb}, *out-of-plane deformation*

Analysis of Raman shifts of exosomes: Because the polarizability of the in-plane stretching of C=O and C-N involved in peptide changes greatly, the amide group located in the plane is expected to produce relatively high Raman intensity. In addition, the vibration of aromatic side chain (phenylalanine, tyrosine, tryptophan) ring is also considered to be strong in Raman spectrum. While the bending and stretching modes of hydrogen like substituents (C-H, N-H, O-H) are usually weak in Raman spectra. However, due to the large number of such groups in proteins or lipids, the collective Raman intensity may be very high.

Table S2 The Raman peak assignment of exosomes

Raman Shift (cm^{-1})	Peak assignment	References
566 cm^{-1}	Carbohydrate present in cell membrane	[S6]
810 cm^{-1}	Histidine, δ (=CH)	[S7]
859 cm^{-1}	Cysteine & Cystine, v_s (CCN)	[S7]
1040 cm^{-1}	Lipid layer components of cell membrane	[S6]
1140 cm^{-1}	Deoxyribose phosphate backbone v (C-C), Adenosine, [illegible]	[S8]
1240 cm^{-1}	Histidine, ρ_{ipb} (Imidazole)	[S7]
1267 cm^{-1}	Tyrosine	[S9]
1300 cm^{-1}	Amide III, v (C-N), δ (N-H)	[S10]
1450-1470 cm^{-1}	Nucleic Acids (DNA or RNA), proteins and lipids, v (C-H), [illegible]	[S11]
2870-2920 cm^{-1}	CH、CH_2&CH_3 of aliphatic group, v(C-H)	[S11, S12]

Abbreviation: δ, deformation; v, stretching; v_s symmetric stretching; b, bending vibrations; $\rho_{ipb,}$ in-plane deformation

S3 SERS Enhancement Factor (EF) Calculation

Based on the equation [S13]:

$$EF = \frac{I_{SERS}}{N_{SERS}} \frac{N_{bulk}}{I_{bulk}} \qquad \text{(S1)}$$

In the formula, EF is the enhancement factor. I_{SERS} and I_{bulk} are the Raman intensity of the fingerprint peaks in the SERS spectrum of the measured molecule and the spectrum measured without SERS substrate enhancement, respectively. N_{SERS} is the average number of molecules to be measured in the SERS scattering cross section, and N_{bulk} is the average number of molecules to be measured in the ordinary Raman scattering cross section.

$$N_{SERS} = \frac{CVN_A A_{Raman}}{A_{substrate}} \qquad \text{(S2)}$$

Where C (mol L^{-1}) is the concentration of the measured molecular solution, V (m^3) is the volume of the solution dropped on the glass slide, and N_A (mol^{-1}) is the Avogadro constant. A_{Raman} and $A_{substrate}$ (m^2) are the area of laser and droplet diffusion on the glass slide, respectively.

$$N_{bulk} = \frac{\rho h N_A A_{Raman}}{M} \qquad \text{(S3)}$$

Where ρ is the density of R6G (g cm^{-3}), h is the focusing depth of laser source (μm), A_{Raman} (m^2) is the laser spot area and N_A (mol^{-1}) is the Avogadro constant.

Among them, $d = 4\ \mu m$, $\rho = 1.1702\ g/cm^3$, $h = 21\ \mu m$, [S14] $N_A = 6.022 \times 10^{23}$, $M = 479.01\ g/mol$

S3.1 EF of BP Nanosheets

The equation (1) can be simplified to:

$$EF = \frac{I_{SERS}}{C_{SERS}} \times \frac{C_{bulk}}{I_{bulk}} \qquad \text{(S4)}$$

For R6G powder,

$$C_{bulk} = \frac{n}{V} = \frac{m}{MV} = \frac{\rho}{M} = \frac{1.1702}{479.01} \times 10^3 = 2.443 \qquad \text{(S5)}$$

So as long as we get C_{SERS}, I_{SERS} and I_{bulk}, the EF of BP nanosheets can be obtained.

S3.2 EF of Ag/Black Phosphorus Nanosheets

Here we calculate the EF of the spectrum of a single R6G molecule, so $N_{SERS} = 1$.

Take the EF of Ag/BP-NS at 1507 cm^{-1} for R6G with 10^{-17} M concentration as an example. As a result，

$$N_{SERS} = 1$$

$$N_{bulk} = \frac{\rho h N_A A_{Raman}}{M} = \frac{1.1702 \times 21 \times 6.022 \times 10^{23} \times \pi \times (2 \times 10^{-6})^2}{479.01} = 3.88 \times 10^{11}$$

$$EF_{1507\ cm^{-1}} = \frac{I_{SERS}}{N_{SERS}} \frac{N_{bulk}}{I_{bulk}} = \frac{1518.5}{1} \frac{3.88 \times 10^{11}}{5828.9} = 0.101 \times 10^{12}$$

S4 The Number of Molecules and Exosomes under Each Raman Window

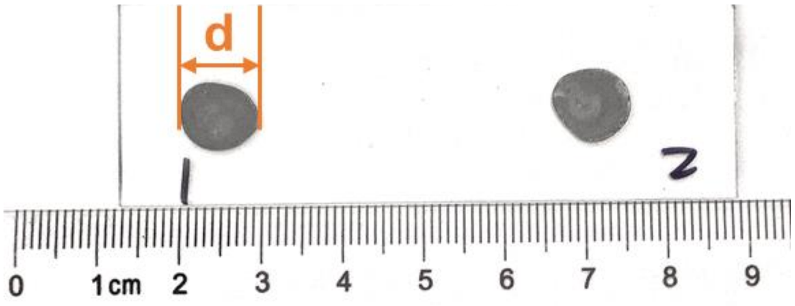

Fig. S11 Spot size of Ag/BP-NS diffusion on glass plate ($d \approx 10\ mm$)

Based on the equation:

$$N = N_{substrate} \frac{A_{Raman}}{A_{substrate}} = \frac{CVN_A A_{Raman}}{A_{substrate}}$$

In the formula, N is the number of molecules or exosomes under each Raman window. $N_{substrate}$ is the number of molecules or exosomes in the droplet on the glass plate. A_{Raman} and $A_{substrate}$ (μm^2)are the area size of laser and droplet, respectively. The diameter of laser spot is 1 μm. C (mol L^{-1}) and V (L) are the concentration and volume of the droplet, respectively. N_A (mol^{-1}) is the Avogadro constant.

Among them,

$$A_{Raman} = \pi r^2 = \pi \times 1^2\ \mu m^2, A_{substrate} = \pi r^2 = \pi \times 5000^2\ \mu m^2,$$

$$N_A = 6.022 \times 10^{23}$$

S4.1 The Number of Molecules under Raman Window

Here, take C = 10^{-11} mol/L, $V = 50\ \mu L = 0.5 \times 10^{-4}\ L$, then

$$N = \frac{1 \times 10^{-11} \times 0.5 \times 6.022 \times 10^{23} \times \pi \times 0.5^2}{10^4 \times \pi \times (5000)^2} = 3$$

S4.2 The Number of Exosomes under Raman Window

Here, take C = 5 × $10^7\ particles/mL$, $V = 100\ \mu L = 0.1\ mL$, then

$$N = 5 \times 10^7 \times 0.1 \times \frac{\pi \times 0.5^2}{\pi \times (5000)^2} = 0.05$$

S5 Photonic Properties Calculation of BP Nanosheets

The photonic properties of a monolayer BP can be described by employing a simple semiclassical Drude model [S15]. The conductivity is given as：

$$\delta_{jj} = \frac{iD_j}{\pi\left(\omega + \frac{i\eta}{\hbar}\right)}$$

Where *j* represents the direction,

$$D_j = \frac{\pi e^2 n}{m_j}$$

is the Drude weight, $\boldsymbol{n}$ is the electron doping concentration, $\boldsymbol{\eta}$ is the scattering rate. The electron masses in the $\boldsymbol{x}$ direction and $\boldsymbol{y}$ direction can be described as

$$m_{cx} = \frac{\hbar^2}{\frac{2\gamma^2}{\Delta} + \eta_c},\ m_{cy} = \frac{\hbar^2}{2v_c}$$

The parameters are determined by fitting the known anisotropic mass. Where $\gamma = \frac{4a}{\pi} eVm$, $\Delta = 2eV$, $\eta_c = \frac{\hbar^2}{0.4m_0}$, $v_c = \frac{\hbar^2}{1.4m_0}$ and $\eta = 10\ meV$. The equivalent dielectric constant of a thin layer using two-dimensional conductivity can be expressed as

$$\varepsilon_{jj} = \varepsilon_r + \frac{i\delta_{jj}}{\varepsilon_0 \omega a}$$

As a result, $\boldsymbol{\varepsilon} = \begin{bmatrix} \varepsilon_x & 0 & 0 \\ 0 & \varepsilon_y & 0 \\ 0 & 0 & 1 \end{bmatrix}$, where $\varepsilon_x = 5.31 + 0.0091i$, $\varepsilon_y = 5.58 + 0.000767i$.

In the FDTD simulations, the BP film thickness is chosen to be 1nm. Because the anisotropic optical properties of BP, we have calculated 5 layers BP (thickness 5 nm) in the X and Y polarizations plane source (532 nm). And the mesh size is 0.25nm. The distance between the particles and BP substrate is 1nm.

S6 Machine Learning Methods

SVM models were trained using toolbox LIBSVM 3.24 for MATLAB [S16]. The training accuracy of Linear kernels, Polynomial kernel and RFB kernel were verified through the cross-validation program, and finally the Linear kernel with the highest accuracy was selected. We can get the difference between A549 exosomes, HCT116 exosomes and PBS by SVM (based on linear kernel function). A total of 933 Raman shifts from 500 to 1550 cm^{-1} were chosen as the variables for SVM using MATLAB. The built-in "SVM" function was used to get principal component coefficients, training set, testing set and hyperplane, etc. Error ellipses of 95%

confidence were plotted by using the "error-ellipse" function. According to SVM, we can get the main difference of tumor exosomes of different cell lines. The trained model achieved values of sensitivity in the prediction of training and testing sets equal to 100 and 99.17%, respectively. There are only one case of misjudgment appeared in the testing set. The results show that we can identify tumor exosomes by Raman spectra combined with SVM based on Ag/BP-NS substrates.

Supplementary References

[S1] Z. Liu, H. Chen, Y. Jia, W. Zhang, H. Zhao et al., A two-dimensional fingerprint nanoprobe based on black phosphorus for bio-SERS analysis and chemo-photothermal therapy. Nanoscale **10**(39), 18795-18804 (2018). https://doi.org/10.1039/c8nr05300f

[S2] H. Wang, X. Yang, W. Shao, S. Chen, J. Xie et al., Ultrathin black phosphorus nanosheets for efficient singlet oxygen generation. J. Am. Chem. Soc. **137**(35), 11376-11382 (2015). https://doi.org/10.1021/jacs.5b06025

[S3] P. Li, W. Chen, D. Liu, H. Huang, K. Dan et al., Template growth of Au/Ag nanocomposites on phosphorene for sensitive SERS detection of pesticides. Nanotechnology **30**(27), 275604 (2019). https://doi.org/10.1088/1361-6528/ab12fb

[S4] W. Chen, J. Ouyang, X. Yi, Y. Xu, C. Niu et al., Black phosphorus nanosheets as a neuroprotective nanomedicine for neurodegenerative disorder therapy. Adv. Mater. **30**(3), 1703458 (2018). https://doi.org/10.1002/adma.201703458

[S5] Y. Zhai, Y. Zheng, Z. Ma, Y. Cai, F. Wang et al., Synergistic enhancement effect for boosting Raman detection sensitivity of antibiotics. ACS Sens. **4**(11), 2958-2965 (2019). https://doi.org/10.1021/acssensors.9b01436

[S6] J. Sundaram, B. Park, A. Hinton, K.C. Lawrence, Y. Kwon, Detection and differentiation of salmonella serotypes using surface enhanced Raman scattering (SERS) technique. J. Food Meas. Charact. **7**(1), 1-12 (2013). https://doi.org/10.1007/s11694-012-9133-0

[S7] E. Proniewicza, A. Tata, M. Starowicz, A. Wojcik, J. Pacek et al., Is the electrochemical or the "green chemistry" method the optimal method for the synthesis of ZnO nanoparticles for applications to biological material? Characterization and SERS on ZnO. Colloid Surf. A Physicochem. Eng. Asp. **609**, 125771 (2021). https://doi.org/10.1016/j.colsurfa.2020.125771

[S8] B. Prescott, W. Steinmetz, G.J. Thomas, Characterization of DNA structures by laser Raman spectroscopy. Biopolymers **23**(2), 235-256 (1984). https://doi.org/10.1002/bip.360230206

[S9] D. Němeček, G.J. Thomas, Raman spectroscopy in virus structure analysis. *Digital Encyclopedia of Applied Physics* (2009). https://doi.org/10.1002/3527600434.eap670

[S10] T. Miura, G.J. Thomas, Raman spectroscopy of proteins and their assemblies. *Proteins: Structure, Function, and Engineering* 55-99 (1995). https://doi.org/10.1007/978-1-4899-1727-0_3

[S11] M. Keshavarz, B. Tan, K. Venkatakrishnan, Label-free SERS quantum semiconductor probe for molecular-level and in vitro cellular detection: a noble-metal-free methodology. ACS Appl. Mater. Interfaces **10**(41), 34886-34904 (2018). https://doi.org/10.1021/acsami.8b10590

[S12] R. Haldavnekar, K. Venkatakrishnan, B. Tan, Non plasmonic semiconductor quantum SERS probe as a pathway for in vitro cancer detection. Nat. Commun. **9**(1), 3065 (2018).

https://doi.org/10.1038/s41467-018-05237-x

[S13] Y.S. Peng, C.L. Lin, M. Tang, L.L. Yang, Y. Yang et al., Niobium pentoxide ultra-thin nanosheets: a photocatalytic degradation and recyclable surface-enhanced Raman scattering substrate. Appl. Surf. Sci. **509**, 145376 (2020). https://doi.org/10.1016/j.apsusc.2020.145376

[S14] L.L. Yang, Y.S. Peng, Y. Yang, J.J. Liu, H.L. Huang et al., A novel ultra-sensitive semiconductor SERS substrate boosted by the coupled resonance effect. Adv. Sci. **6**(12), 1900310 (2019). https://doi.org/10.1002/advs.201900310

[S15] Z.Z. Liu, K. Aydin, Localized surface plasmons in nanostructured monolayer black phosphorus. Nano Lett. **16**(6), 3457-3462 (2016). https://doi.org/10.1021/acs.nanolett.5b05166

[S16] C.C. Chang, C.J. Lin, LIBSVM: a library for support vector machines. ACM Trans. Intell. Syst. Technol. **2**(3), 27 (2011). https://doi.org/10.1145/1961189.1961199
